# Based on a Single Case, Cardiopulmonary Training Cannot Be Recommended as Therapy for Hemiparesis in MELAS With Ischemic Stroke

**DOI:** 10.1002/kjm2.70294

**Published:** 2026-09-18

**Authors:** Sounira Mehri, Josef Finsterer

**Affiliations:** ^1^ Biochemistry Laboratory, LR12ES05 “Nutrition‐Functional Foods and Vascular Health”, Faculty of Medicine Monastir Tunisia; ^2^ Neurology & Neurophysiology Center Vienna Austria

To the Editor,

1

We read with interest the article by Tsai et al. on a 49‐year‐old man with mitochondrial encephalopathy, lactic acidosis, and a stroke‐like episode syndrome (MELAS) caused by the MT‐TL1 variant m.3243A>G, which manifested as bilateral hearing loss, diabetes, hyperlipidemia, and left hemiparesis resulting from an ischemic stroke (IS) shortly after strength training [1]. Following an inpatient rehabilitation program with regular cardiopulmonary exercise testing (CPET) over 12 weeks, the patient made a largely full recovery [1]. The study is insightful but warrants further discussion.

First, there was no report on how IS was distinguished from stroke‐like episodes (SLE) [1]. Did the right‐sided lesion in the brainstem actually meet the criteria for an IS? Was the patient treated with thrombolysis or thrombectomy? What other risk factors for an IS were present besides diabetes and hyperlipidemia? It was not reported whether the patient suffered from arterial hypertension, atrial fibrillation, heart failure, a coagulation disorder, or a hematological disease, or whether he was a smoker. Was a dissection of the vertebral artery or a cardioembolism definitively ruled out? An IS in MELAS is often confused with a SLE, which is characterized by a distinct clinical and imaging presentation as well as a variable course of the IS. SLEs on imaging are characterized by hyperintensity on T2/FLAIT, DWI, and PWI, as well as hypointensity on OEF‐MRI known as stroke‐like lesion (SLL). In addition, FDG‐PET shows marked hypometabolism in the area of a SLL. Furthermore, the dynamics of a SLL differ significantly from those of an IS, and the lesion volume does not correspond to a vascular territory. In SLLs, DWI may be hyperintense and the ADC hypointense only in the cerebral cortex, while the ADC may exhibit variable intensity in the remainder of the SLL region [2].

Second, given the study design (case report), it cannot be ruled out that the improvement attributed to the exercise training actually corresponds to the natural course of the disease. Case reports have several drawbacks. Among their greatest disadvantages are the inability to establish cause‐and‐effect relationships (a case report cannot conclusively prove that a specific treatment caused a particular outcome); they are subject to publication bias and overinterpretation of coincidental events; they lack statistical validity; and there is an inherent inability to generalize the results to broader populations [3].

The third point is that it was not discussed that an mtDNA mutation can phenotypically manifest as primary mitochondrial vasculopathy [4]. Vasculopathy in mitochondrial diseases can manifest as arterial dissection, atherosclerosis, aortic rupture, ectasia, and aneurysm formation [4].

The fourth point is that several pieces of information are missing to assess whether the CPET or the natural course of the disease was responsible for the outcome, and whether the index patient actually suffered an IS or rather an SLE, a pathognomonic feature of MELAS. For example, the heteroplasmy rate, a key determinant of the phenotypic expression of the m.3243A>G variant, is missing, as is a measurement of cerebral lactate.

Overall, the conclusions drawn are not supported by the data presented. A single case cannot demonstrate the efficacy of a treatment. In particular, it is not justified to generalize and extrapolate the results of a single patient to the entire population of patients with mitochondrial disorders. CPET cannot be recommended as a standard therapy for all patients with impaired aerobic oxidative metabolism until valid studies confirm this effect, and treating physicians should refrain from prescribing CPET as a general treatment for all patients with a mitochondrial disorder and IS.

## Ethics Statement

This article is based on previously conducted studies and does not contain any new studies with human participants or animals performed by any of the authors.

## Conflicts of Interest

The authors declare no conflicts of interest.

## Data Availability

The data that support the findings of this study are available on request from the corresponding author. The data are not publicly available due to privacy or ethical restrictions.
